# A Novel Makeshift Method to Collect Leaking Epidural Fluid: A Case Report

**DOI:** 10.7759/cureus.60553

**Published:** 2024-05-18

**Authors:** Kimmy Bais, Vikas Raghove, Hattiangadi Sangeetha Kamath, Joel Yarmush

**Affiliations:** 1 Anesthesiology, The Ohio State University Wexner Medical Center, Columbus, USA; 2 Anesthesiology, Indiana University (IU) Health Ball Memorial Hospital, Muncie, USA; 3 Anesthesiology, NewYork-Presbyterian Brooklyn Methodist Hospital, Brooklyn, USA

**Keywords:** epidural catheter, interstitial fluid, csf composition, postoperative cerebrospinal fluid leak, labor epidural analgesia

## Abstract

A healthy 34-year-old full-term parturient was admitted to the labor suite where a combined spinal-epidural (CSE) was easily placed on the first attempt for labor analgesia. After an uneventful delivery, the epidural catheter was removed. Two days later, the patient experienced a fluid leak from the puncture site. The fluid was analyzed to determine whether it was an interstitial or a cerebrospinal fluid (CSF). We describe a novel technique to collect the leaking fluid without admixing fluid from the surrounding area. No previous reports describe a similar technique to diagnose the source of this questionable fluid leak.

## Introduction

An epidural procedure involves seating an epidural needle in the epidural space and placing an epidural catheter there for the continuous administration of medication before removing the epidural needle. A combined spinal-epidural (CSE) involves placing a large gauge (G) spinal needle through the epidural needle and continuing beyond the epidural space through the dura and into the subarachnoid space. Intrathecal medication is administered through the spinal needle, which is then removed, and the epidural catheter is placed before removing the epidural needle. The cerebrospinal fluid (CSF) leaking through the very small dural puncture hole is uncommon [1]. It is even rarer that the CSF leak would extend out through multiple layers of the skin [1]. If there is a fluid leaking out of the skin, it is generally more likely to be interstitial fluid. However, it becomes necessary to rule out CSF leak because of the associated risk of central nervous system infection [1-3], hematoma, intracranial hemorrhage, post-dural puncture headache, and even brain herniation. A fluid leak out of the skin may be seen immediately upon the removal of the epidural catheter or at some later time [1-4]. The biochemical composition of CSF and interstitial fluid is very different, so the biochemical analysis may prove helpful to differentiate one from another. We present a case involving a post-CSE fluid leak and describe a method for fluid collection in an aseptic manner. Health Insurance Portability and Accountability Act (HIPAA) authorization has been obtained in writing from the patient.

## Case presentation

A healthy 34-year-old American Society of Anesthesiologists (ASA) physical status 2 full-term gravida 2, para 2 parturient received a CSE block at the L3-L4 intervertebral level for labor analgesia. The block was performed utilizing a 17 G 9 cm-long Tuohy needle and a Luer slip glass syringe filled with saline. The epidural space was identified successfully on the first attempt at a depth of 6 cm from the skin with the loss of resistance technique. A 25 G Gertie Marx (pencil tip, 12.8 cm long) spinal needle was placed through the Tuohy needle into the subarachnoid space, and 2 cc of the standard epidural drug solution (0.0625% bupivacaine and 2 mcg/cc fentanyl) was administered intrathecally to provide quick pain relief. Following this, the epidural catheter was threaded through the Tuohy needle in the first attempt and placed 12 cm deep into the skin. There was no CSF or blood aspirated through the catheter. Labor epidural analgesia was maintained with continuous epidural infusion at 12 cc/hour along with patient-controlled epidural boluses. The patient endorsed adequate pain relief with epidural analgesia. A healthy baby was delivered after seven hours of uneventful labor. The epidural catheter was subsequently removed with the tip intact. No leak was noted at that time. The patient was transferred to the mother and baby unit.

On the second postdelivery day around 24 hours after epidural catheter removal, the patient complained that her clothes in the back felt wet and soaked. The anesthesiology team was consulted for suspicion of a possible CSF leak. On the examination of the back, the epidural site dressing was noted to be saturated with clear fluid. The dressing was removed, and clear fluid droplets were found draining from a flat non-edematous pinhole-sized skin puncture site. The patient had no complaints of headache, photophobia, double vision, sensory/motor weakness, or localized back tenderness. She was alert and oriented, afebrile, and able to ambulate with an appropriate level of assistance after delivery. The neurological examination was insignificant. The laboratory results in the patient’s chart revealed no anemia, leukocytosis, or hypoalbuminemia. We suspected interstitial fluid but needed to rule out a CSF leak. The patient had an uneventful delivery other than the fluid leak and was a candidate for discharge after 48 hours. It was planned to collect the leaking fluid aseptically and get a biochemical analysis of the fluid to rule out the CSF leak and help with quick discharge for the patient.

To collect the fluid aseptically for analysis, the puncture site and the surrounding skin were first cleaned with chlorhexidine. A makeshift apparatus was applied to the puncture site with sterile gloved hands, and the fluid was allowed to collect in a small round reservoir under gravity.

**Figure 1:**
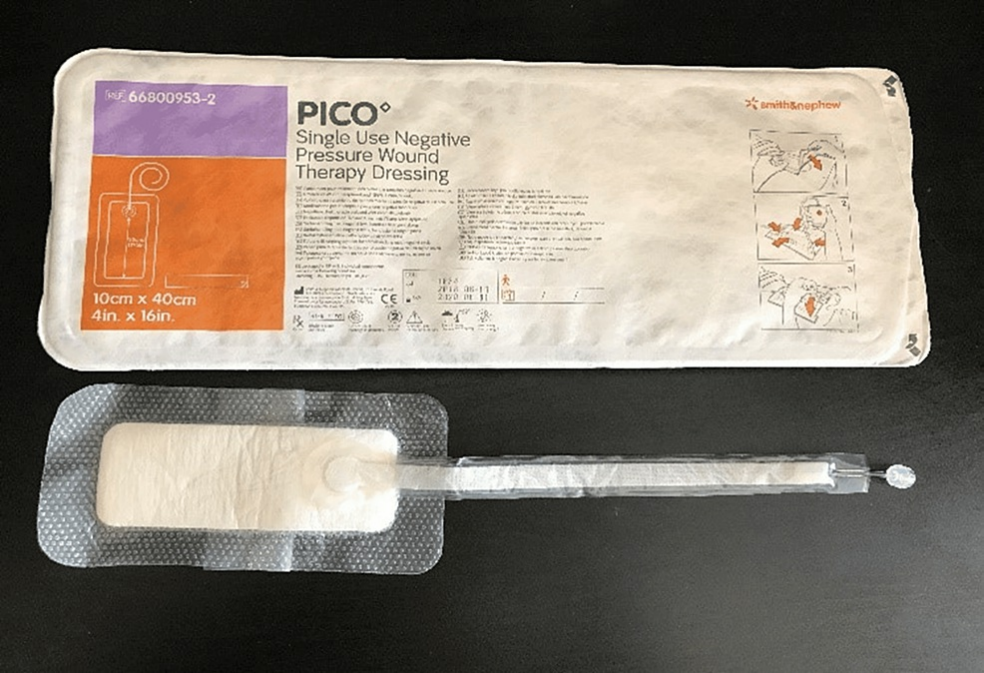
PICO Single Use Negative Pressure Wound Therapy Dressing

**Figure 2:**
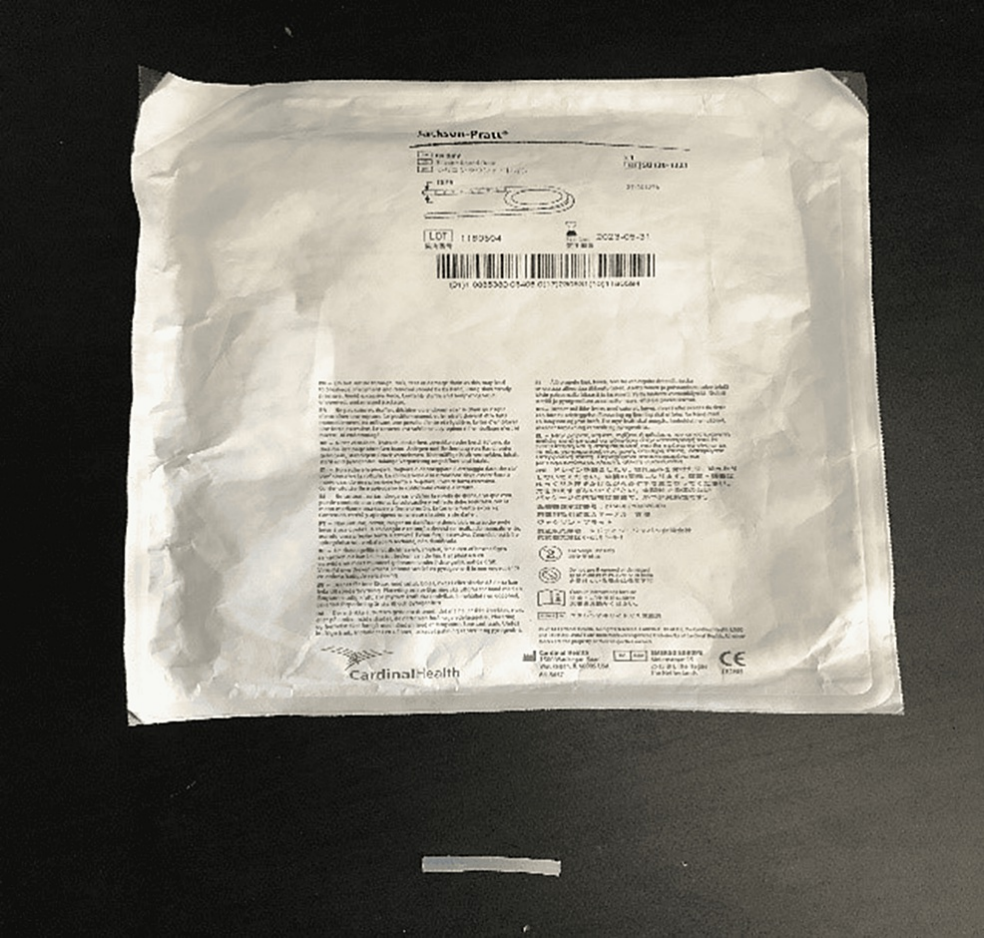
Jackson-Pratt 10 Fr silicon round drain

**Figure 3:**
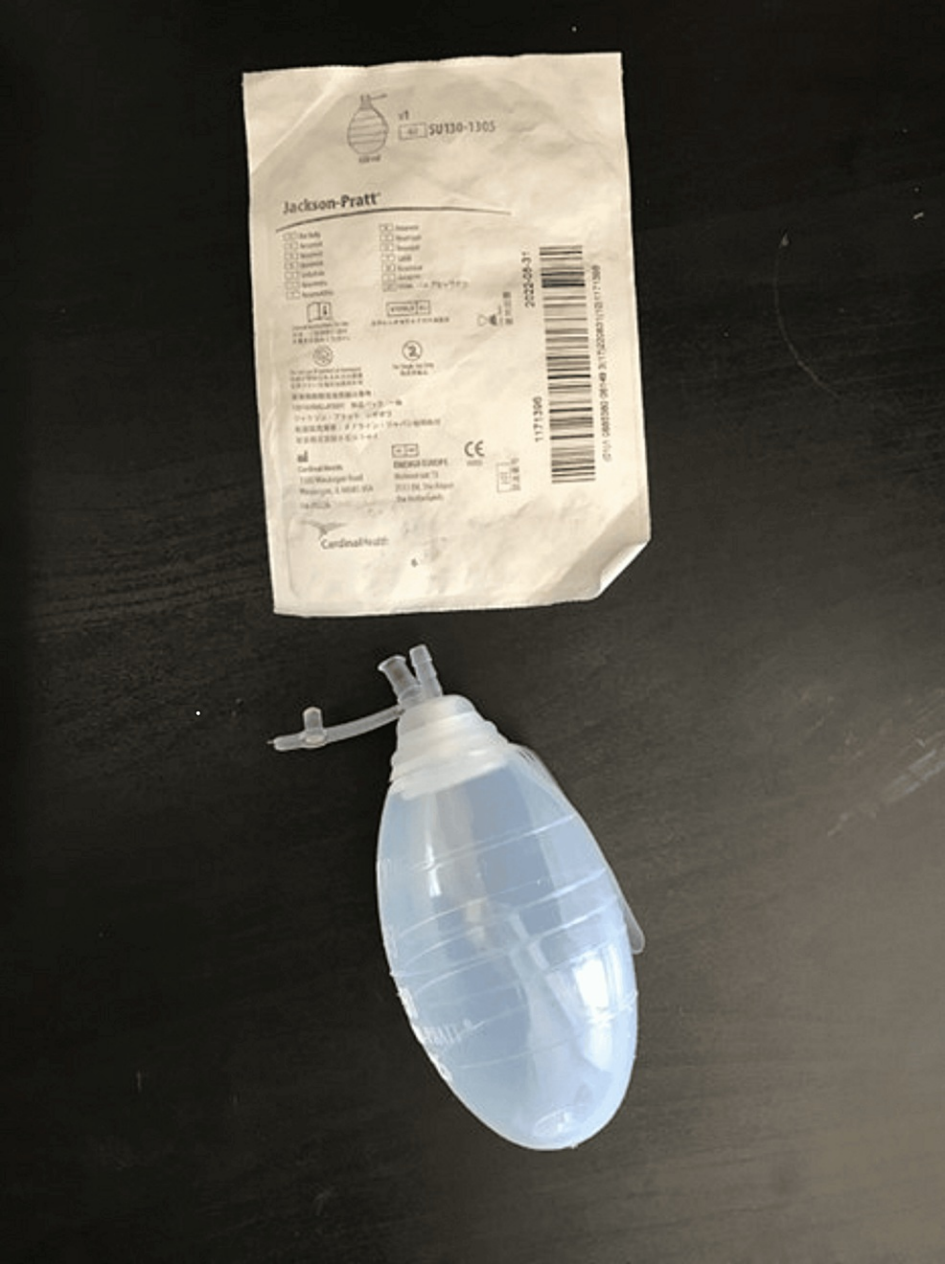
Jackson-Pratt 100 cc reservoir

The apparatus consisted of three parts: (a) PICO Single Use Negative Pressure Wound Therapy Dressing and the sealing tape provided with the dressing (Figure 1), (b) 10 Fr Jackson-Pratt silicon round drain tubing connector (Figure 2), and (c) 100 cc Jackson-Pratt reservoir with ejection hole (Figure 3).

**Figure 4:**
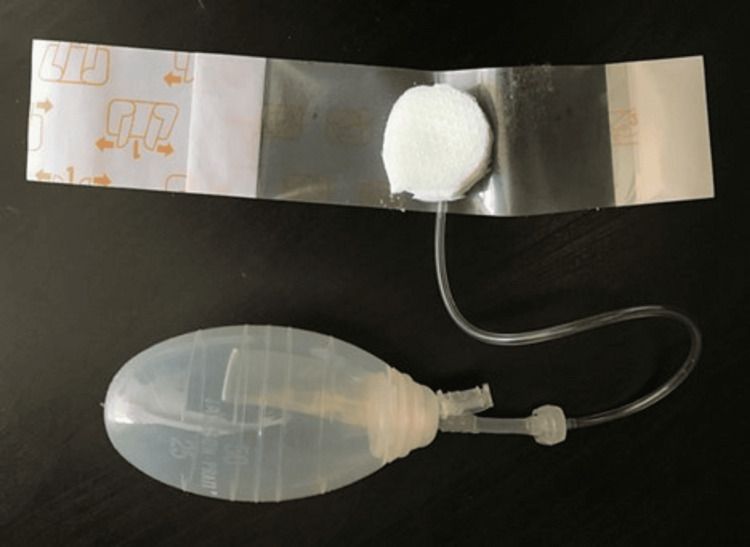
The assembly with the PICO dressing cut into a circle with the hub intact and the sealing tape surrounding it

With sterile gloved hands on a sterile draped table, we assembled the apparatus by connecting the distal hub of the tubing of PICO dressing with the small connector, which comes attached to a “10 Fr Jackson-Pratt silicon round drain.” Then, the connector’s distal end was further connected to the “100 cc Jackson-Pratt reservoir.” We cut the PICO dressing into a smaller-sized circular dressing with the suction hub intact (Figure 4). The reservoir was squeezed, and the ejection hole was sealed with the attached plug to create negative pressure. The assembly was secured well, and the fluid was allowed to drain into the reservoir under gravity. We collected 10 cc of sterile fluid in the next three hours. The fluid was sent for cytology, culture, and biochemical analysis to the laboratory. The results revealed that the chemical composition of the collected fluid was closer to interstitial fluid. The CSF has lower glucose, partial pressure of carbon dioxide (pCO_2_), and K+ as compared to the interstitial fluid [5].

Since the fluid composition was found to be similar to interstitial fluid, conservative measures were taken, and a pressure dressing was applied to the puncture site along with an abdominal binder. The neurosurgery team examined the patient and ruled out any urgent neurological intervention. The leak resolved on the third postoperative day, and the patient was cleared by all teams to be discharged home.

## Discussion

A leak at the epidural puncture site, whether it is from the CSF or interstitial fluid, is an infrequent occurrence. While more likely interstitial, it may be CSF, and that would be concerning. The incidence of CSF leak increases with multiple dural punctures with large bore needles [6,7] and undiagnosed connective tissue disorders. The clinical examination, neurological assessment, and temporal association of events [1] can be indicative but are not confirmatory. Various tests to differentiate the two include the biochemical analysis of the fluid, protein electrophoresis for the presence of CSF-specific acetylcholinesterase, β2-transferrin immunofixation (specific for CSF), and radioisotope myelography to look for a CSF fistula. Biochemical analysis is a quick and economical option and would be reliable provided that the fluid is not mixed with any other bodily material from the skin or surrounding structures, which can alter the biochemical laboratory values. This means that a sterile technique for specimen fluid collection is the key to the diagnosis.

One collection technique has been described by Dalal and Shrividya [8] using an attached colostomy bag. The colostomy bag would be appropriate if the leakage is significant and the rate of leak is fast enough to allow fluid collection for different biochemical tests. The insertion of a hypodermic needle in the subcutaneous tissue away from the epidural leak site and the collection of similar composition fluid from the subcutaneous tissue have also been tried in the past to rule out CSF leaks [8,9].

In our apparatus, cutting the PICO dressing to the smallest possible size prevented unnecessary contact with the surrounding skin and environment, thus decreasing the chance of admixing and providing a tight seal at the same time. The attached Jackson-Pratt reservoir bag allowed the creation of a slight negative pressure, which aided in the collection of fluid under gravity. If the values are equivocal, more complex, expensive, and time-consuming tests as delineated above are available.

Our patient was a healthy young parturient, with no medical problems and nontraumatic single-pass CSE placement. Our purpose to present the collection method was to obtain a quick economical answer when the suspicion is low to enable the timely discharge of the patient.

## Conclusions

CSF leak from the epidural skin puncture site is concerning and should be ruled out to prevent neurological complications. We describe a novel makeshift method of collecting fluid aseptically from the puncture site, which can result in minutes for biochemical analysis. This might help diagnose the cause of fluid leak quickly and avoid unnecessary discharge delay and hospital expenses in cases with low suspicion. Further research is required to study the reliability and reproducibility of this technique and compare it with any upcoming methods.
